# Czech version of OPQOL-35 questionnaire: the evaluation of the psychometric properties

**DOI:** 10.1186/s12955-016-0494-7

**Published:** 2016-06-18

**Authors:** Jiri Mares, Hynek Cigler, Eva Vachkova

**Affiliations:** Department of Social Medicine, Charles University in Prague, Faculty of Medicine in Hradec Kralove, Hradec Kralove, CZ Czech Republic; Department of Psychology, Masaryk University, Faculty of Social Sciences in Brno, Brno, CZ Czech Republic

**Keywords:** Seniors, Quality of life, Assessment, OPQOL-35 questionnaire, Factor structure, Reliability, Validity

## Abstract

**Background:**

Both prognoses and real demographic trends in developed countries point to the increasing proportion in the population of people above 65 years of age. One of important themes of care for seniors is the assessment of their quality of life. To evaluate the quality of life of seniors three types of tools can be used: general generic tools; generic tools used for the age group of elderly persons; specific tools to detect the quality of life of the elderly who are affected by specific diseases.

**Methods:**

The second type of tool is represented by the OPQOL - 35 questionnaire (*Older People’s Quality of Life* Questionnaire), which was developed in the UK. It has 35 items and deals with 8 domains of quality of life. With the consent of the author the questionnaire was translated into Czech and verified in a group of 478 seniors aged 60 and above (40 % males, 60 % females). Unlike the British version, the Czech version has seven factors: 1 Role of belief, religion and culture; 2 Health; independence, active life; 3 Financial situation; 4 Family and safe environment; 5 Loneliness; 6 Satisfaction with life; 7 Positive approach to life.

**Results:**

The Czech version has a very good reliability (Cronbach’s alpha ranges from .726 to .905). It also has satisfactory validity. The results show that with increasing age and number of health problems the satisfaction of the elderly is declining in all seven domains. Conversely, the degree of autonomy in the way of living is positively associated with the satisfaction of seniors. Old people who live alone at home, are self-sufficient and do not need the help of others, are more satisfied with their quality of life than other seniors (i.e..those who are living with their children, in sheltered accommodation or in homes for the elderly). Single, married seniors and seniors with a partner are happier than the widowed ones.

**Conclusions:**

The questionnaire gives good guidance for assessing the current state of the quality of life of seniors, changes in quality over time and for targeted interventions as well.

## Background

### Introduction

Both prognoses and actual demographic trends in developed countries point to the increasing proportion in the population of people above 65 years of age. While seniors over 65 made up 11 % of the population of the European Union in the year 1960, an increase to 22 % is expected in 2020, and the number will keep on rising in following years [1]. The middle age of both males and females becomes longer, and this leads to thinking how far the “added years” of seniors will be spent in relative comfort, how far their needs will be met.

The ageing society itself should provide an opportunity to work for all seniors who want to lead an active way of life. Health care must be designed so as to prevent long-term dependence on institutional care of the elderly in dealing with their health problems. Seniors should be given the option of an active and independent life in old age, not only be provided with an expanding network of residential facilities [2]. Hence, one of the important themes of care for seniors is their quality of life.

### Quality of life in old age

Main domains characterizing the quality of human life that now appear in the literature are common for adults of different ages. According to Arnold they include: the cognitive, emotional, social, sexual, and behavioural functioning as well as perceived social support, life satisfaction, assessment of one’s own health, assessment of one’s own economic situation, the level of satisfaction of interests, one’s energy and vitality [3]. Experts add that in adulthood the areas are the same, nevertheless at different stages of life the emphasis is shifting to other areas, the priorities are different. What does it look like in old age?

The key factor in our further considerations is the fact that quality of life is understood as a matter of subjective and not objective data. In other words, it depends on the individual’s own unique perception, experience and evaluation of individual domains of quality of life, not on “what they are objectively like”, as they appear to outside observers. For the quality of life in old age the following domains are particularly important: health condition and functional ability of the senior, sense of being useful/useless for other people, network of social relationships, perceived level of social support, financial situation, and the quality of housing [4].

Why do we encounter different domains of quality of life in various papers on the elderly? It turns out that there are various models of quality of life used by different authors. In the very profound survey work [4], we counted ten of them, but the ways of sorting them intersect (according to the nature of indicators, to scientific discipline or according to methodological approaches). In principle it is possible to distinguish four groups of models: 1st psychological, 2nd sociological, 3rd medical, 4th integrated. These models were developed and validated by experts and professionals.

The afore-mentioned models therefore represent the view of professionals on what constitutes the quality of life. Equally important, however, is the view from the other side, the view of laymen. Seniors especially view themselves according to what constitutes the quality of life to them, and what their priorities are. Bowling and Gabriel carried out an investigation of a representative sample of 999 British seniors aged 65 and more [5]. They used a mixed approach. At first they used the personalized questionnaire SEIQOL-*Schedule for the Evaluation of Individual Quality of Life* [6]. In it, the individuals have the option to choose five themes of life, life goals, on which their life depends the most and then evaluate how much they succeed in fulfilling them. The questionnaire was supplemented with in-depth interviews with 80 senior citizens, which were recorded and then analyzed. In the cited British research the following life themes from the perspective of seniors appeared: social relationships and close interpersonal relationships (81 %); social roles and social activities (60 %); leisure activities that make the individuals happy (48 %); health (44 %); positive attitude to life and feeling of well-being, good mental functioning (38 %); home and neighbours (37 %); financial situation (33 %); autonomy and independence from other people (27 %).

One of the paradoxes of diagnosing the quality of life in old age is – as mentioned by [3]– that quality of life is intuitively understood as a positive or at least neutral phenomenon, but routine diagnostic procedures in the elderly are built mainly on *negative* indicators: on inability, not being self-reliant, on disease, depression, loneliness etc. Only the movement called “positive psychology” drew attention of researchers also to a thorough scientific investigation of the positive aspects of ageing, for example wisdom [7] humour and life satisfaction [8], and others.

Diagnosing the quality of life of elderly people serves primarily five purposes [9]. It allows you:

1. to understand the causes and consequences of individual differences in quality of life,
2. to improve clinical decision-making in patients of the given age group,
3. to assess the effectiveness of health interventions and/or to assess the quality of the health care system,
4. to assess the impact of social and environmental interventions on quality of life,
5. to estimate the needs of the population groups.

Usually, diagnosing the quality of life is characterized according to applied methods as qualitative, quantitative or mixed approaches. We have concentrated on the quantitative approach, which is based on questionnaires that are filled by seniors themselves. We have left aside the generic questionnaire SF-36 or EuroQol Five-Dimension Questionnaire [10], which are not aimed only at seniors, we were interested in questionnaires designed primarily for seniors. There are six of them. If we sort them by the year of their origin, we get this order: CASP-19 – *Control, Autonomy, Self-Regulation, Pleasure* [11], WHOQOL-OLD – *World Health Organization Quality of Life – Old* [12], QUAL-E – *Quality of Life at the End of Life* [13], EQOLI – *Elderly Quality of Life Index* [14, 15], OPOQOL - *Older People’s Quality of Life Questionnaire* [16], ASCOT – *Adult Social Care Outcomes Toolkit* [17]. When considering which one to choose, we have ruled out three of them at once: ASCOT questionnaire because it is designed primarily to assess the social, not medical care; EQOLI questionnaire, the development of which has not yet been completed and the questionnaire QUAL-E, because it focuses on specific period - the quality of life at the end of life. Of the three remaining, i.e. CASP-19, WHOQOL-OLD and OPQOL, the best results in the scientific comparison were reached by the last one [18].

In the questionnaire OPQOL-35 we appreciated that it helps evaluate the quality of life of seniors in general, regardless of their health condition. The OPQOL has excellent applicability to cognitively normal subjects, and it proved to be applicable to people suffering from mild to moderate dementia [19]. It investigates eight domains, thus allowing deliberate intervention with proper knowledge of the facts. We were pleased that OPQOL-35 had acceptable levels of reliability and validity in British population samples of older people, more modest in the ethnically diverse population sample. The questionnaire has potential for use as a multidimensional population survey instrument for use with older populations or as an outcome measure of multisector policy [16].

As a matter of fact, we need to assess the quality of life of both the relatively (for their age) healthy elderly and of those treated on an outpatient basis, but also of the elderly hospitalized at various clinics in the University Hospital. We are interested in the quality of life of seniors, who are living in different social conditions: at home, with family members, in a nursing home, and living in a home for the elderly.

## Methods

### The original version of the OPQOL questionnaire

The OPQOL-35 questionnaire was developed by Ann Bowling of *University College London*, London, Great Britain. The full name of the questionnaire reads: *Older People’s Quality of Life Questionnaire* [5, 16, 18, 20]. The questionnaire is designed to diagnose the quality of life of seniors. In 2008 it was verified in a group of 987 English seniors aged 65 plus. The questionnaire is based on the assumption that quality of life is a multidimensional concept and in the original version allows the evaluation of the quality of life related to eight domains: 1 life overall (4 items), 2 health (4 items), 3 social relationships and participation (8 items), 4 independence, control over life and freedom (5 items), 5 home and neighbourhood (4 items), 6 psychological and emotional well-being (4 items), 7 financial circumstances (4 items), 8 culture and religion (2 items).

The assumption of eight-factor structure is based on seniors’ responses to a short survey with open-ended questions about quality of their life [21]. Responses were coded to eight dimensions and then examined independently by two researchers to inform the inclusion of the items within subscales. The initial pool of 100 items was based on these responses. The selected 49 items were administered to 179 seniors and following analyses reduced their number to 32. Three items (focused on religion and children) were additionally added after a pilot test on a different culture [21].

Nevertheless, factor structure is unclear. Actual authors of OPQOL reported mostly two [22], four [22] or nine-factor solutions [18] based on principal component analysis (PCA). Other authors reported different number of factors, Chen extracted eight factors using PCA, which were similar to the original version [23]; but in our opinion, this solution had methodological lack. No other factor analyses are available. We agree with Ann Bowling [18], author of OPQOL, that a more detailed examination of the factor structure (probably confirmatory) is needed.

The OPQOL questionnaire consisted of 35 statements, with the participants being asked to indicate the extent to which they agree with each statement by selecting one of five possible options (“strongly disagree”, “disagree”, “neither agree nor disagree”, “agree”and “strongly agree”). The range in the original version is based on the principle of point allocation (1–5). Thus – the higher the score, the better the quality of life.

Cronbach’s alphas for the OPQOL-35 in all three samples satisfied the .70–.90 threshold for internal consistency [24]: Cronbach’s alpha .748 (British Ethnibus survey), alpha .876 (British ONS Omnibus survey), alpha .901 (Quality of life follow-up survey).

### The Czech version of the OPQOL questionnaire

With the consent of Ms. Ann Bowling, the author of the original version, the OPQOL-35 questionnaire has been translated into Czech by E. Vachkova and J. Mares. The first step was a literal translation, in the second step the formulations were adjusted to reflect Czech socio-cultural traditions, and each item to be clearly related to its domain at the same time. In the third step, we submitted the text of the questionnaire to 15 seniors and asked them to mark words and formulations that were unclear to them, or could be understood in several ways, or the idea was expressed in a too complicated way. Based on their comments, we modified the text of the questionnaire so that it might be understandable for any elderly person. In the Czech version, we have reversed the five grade scale. We found that our seniors would prefer to evaluate statements using the Czech school grading, because in elementary and secondary schools one is the best mark and five the worst one. The original questionnaire used the principle of point allocation, where five points mean “strongly agree” and one means “strongly disagree”, which confused our seniors.

As we declared earlier, the structure of the original version of OPQOL is unclear, with no reliable factor analysis. The structural equivalence cannot be tested; however, we used a confirmatory factor analysis to verify the original eight-factor model. As the model did not fit (which we expected due to other PCA analyses with different results, [18, 21–23]), we used the ordinal exploratory factor analysis. We consider our approach superior compared to the original PCA. It could help us better understand the factor structure of the OPQOL questionnaire.

Population sample and methods of its selection: We have set the following criteria for the selection of people – aged sixty years or more, good cognitive abilities, seniors of both sexes. The selection was determined by the availability of older people and their willingness to cooperate. They were seniors who, at the time of the research, were hospitalized in the University Hospital in Hradec Kralove (Departments of Cardiology and Angiology, Gastroenterology and Gerontology and Metabolic Diseases) or in smaller urban hospitals, or hospitals for long-term care, or patients treated on an outpatient basis at the general practitioners. Regarding the type of housing, there were the following categories: senior lives alone in his/her apartment and does not need the help of others; senior lives alone, but is occasionally helped by relatives; senior lives with the family; senior lives in sheltered housing or in a nursing home.

Data were collected by selected and instructed sisters of the medical devices.

The complete sample size was 478 people, 192 men (39.8 %) and 246 women (59.3 %); Age: 212 respondents (44.4 %) were aged 60–69 years, 165 (34.5 %) 70–79 years, and 101 (21.1 %) were older than 80.

Though men and women did not differ in their median age, Mann–Whitney *U =* 27350.0*, p =* .994, they differed in their marital status, *χ*^2^(4, *N* = 478) = 19.31, *p* < .001. Using post-hoc Z-test we discovered that men were more often married, z = 5.85, *p* < .001, and women were more often widowed, *z* = -4.23, *p* < .001, see the Table 1 (both tests were significant after Bonferroni’s correction).

**Table 1:** Distribution of sample by sex and marital status

		Sex		
		Males	Females	Total
Marital status	Single	7 (3.6 %)	9 (3.1 %)	16 (3.3 %)
	Married	108 (56.3 %)	106 (31.1 %)	214 (44.8 %)
	Living with a partner	11 (5.7 %)	16 (5.6 %)	27 (5.6 %)
	Divorced	18 (9.4 %)	41 (14.3 %)	59 (12.3 %)
	Widowed	48 (25.0 %)	114 (39.9 %)	162 (33.9 %)
Total		192	286	478

Two hundred fifty-six respondents (53.1 %) were living alone at home, 86 (17.8 %) at home but with other people’s help, 36 (7.5 %) were living with their family out of their own household. Only 16 respondents (3.3 %) were living in a nursing home and 88 (18.3 %) in a retirement home or residential care home. The differences between men and women in the way of living tested by chi-square were not significant, *χ*^2^(4, *N* = 478) = 3.82, *p* = .43.

Only 56 respondents (11.6 %) considered themselves healthy, 319 (66.2 %) had age-appropriate diseases and 107 (22.2 %) were “fairly ill”. Differences between men and women were not significant, *χ*^2^(2, *N* = 478) = 3.56, *p* = .17.

At the end of the questionnaire, respondents could assess their overall quality of life using the five-point Likert scale from 1 (very good) to 5 (very bad). The mean score was 1.46, the differences between men and women were not significant, Mann–Whitney *U* = 27023.0, *p* = .832.

We excluded respondents with missing data in the following item analysis, thus the final sample size was *N* = 458.

## Results

### Psychometric analysis

#### Confirmatory factor analysis

We ran confirmatory factor analysis with correlated eight-factor structure, as was expected in the original version of OPQOL. We used DWLS estimator appropriate for ordinal data with robust error estimates (scaling factor 1.159), no residual correlations were allowed. As the “Religion/culture” factor contained only two items, their loadings were fixed to the same value to prevent negative error variance. This model did not fit the data, *χ*^2^ = 2755.4, *df* = 533, *TLI* = .895, *RMSEA* = .096 (*CI*_*95%*_ = .092 – .099). We also have to consider that items are placed to the questionnaire in the factor order, what should strengthen the expected factor structure, so that we can conclude the actual eight-factor structure is not appropriate.

#### Number of factors

**Fig. 1:**
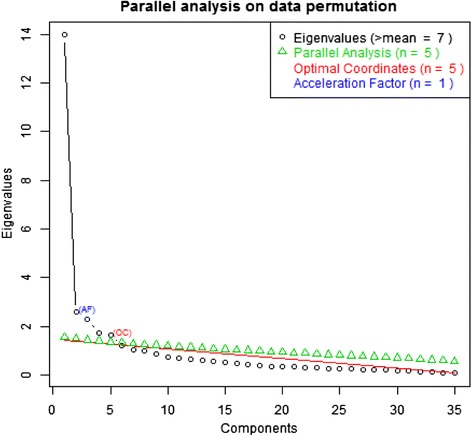
Scree-plot with OC, PA and AC analyses

We estimated the number of factors for extraction in factor analysis. Using the Kaiser criterion, we should have extracted 7 factors with eigenvalue bigger than one, visual control of Cattel scree-plot (Fig. 1) indicated 5 or 6 factors. As these methods are not adequately reliable e.g. [25], we used more advanced techniques as well.

In addition to the basic scree-plot, there are results of “optimal coordinates” (OC) analysis [26] and Horn “parallel-analysis” (PA) [27] at Fig. 1. We used the matrix of polychoric correlations (two-steps), and the optimal number of factors was five. Both these methods are derived from Kaiser’s rule and minimize the impact of random variance in data in different ways. According to the analysis by [28], these methods are highly reliable. In their simulation study, OC was accurate in 74 % with a tendency to underestimate the number of factors, PA in 76.4 % with no significant bias direction. Besides OC and PA, there is also a result of “Acceleration Factor” analysis [26] at the Fig. 1, which indicates the existence of the first order factor.

Finally, we used [28] Comparison Data Analysis using Spearman rank-order correlations, CDR_S_, which showed the best results in their own simulation study (87 % accuracy with a slight tendency to underestimate the factor number, but they used always fewer than five factors). CDR_S_ improves Horn Parallel Analysis and takes also into account sampling error and multivariance data distribution. The optimal number of factors seems to be seven (*p* < .05, see Table 2).

**Table 2:** Comparison data analysis, Ruscio and Roche [28]

Number of factors	*RMSR eigenvalue*	*p-*value
1	.307	.000
2	.289	.000
3	.170	.000
4	.152	.000
5	.122	.000
6	.104	.000
7	.094	.000
8	.095	.201
9	.086	.000
10	.084	.211
11	.087	.789

#### Factor solution

Based on previous results, a series of factor analyses was performed using SPSS 22 [29] with plugin rFactor v2.2.1 [30]. As strong intercorrelations of factors could have flawed the OC and PCA analyses more than CDR_S_, we chose the seven-factors solution, which was also the most meaningful.

We analyzed the matrix of polychoric correlations (two-step estimate) using maximum-likelihood estimation with Kaiser normalization and oblique Geomin rotation. Data were appropriate for factor analysis, KMO = .904 and Bartlett’s test of sphericity *χ*^2^(595) = 12183.3, *p* < .001.

Seven factors explained 62.3 % of variance, of which the first factor explained more than half of it (38.3 %). The factor model fitted data very well, *GFI* = .966, *RMSR* = .032, *RMSP* = .078, and only 9.7 % of residues were higher than .05.

We grouped the factors as follows: 1 Role of belief, religion and culture; 2 Health; independence, active life; 3 Financial situation; 4 Family and safe environment; 5 Loneliness; 6 Satisfaction with life; 7 Positive approach to life.

Table 3 includes factor loadings after rotation (pattern matrix), and inter-correlations of factors are in Table 4. The factor structure is clear and items are loaded mostly by one factor only. The exceptions are items 1, 2 and 3, which are loaded by factor 2 (Health, independence, active life) and 6 (Satisfaction with life), although these factors correlate relatively slightly (*r*_*26*_ = -.176).

**Table 3:** Pattern matrix

Number and wording of the item		F1	F2	F3	F4	F5	F6	F7
		Role of belief and religion	Health, independence, active life	Financial situation	Family and safe environment	Loneliness	Satisfaction with life	Positive approach
34	Religion, belief or philosophy are important to my life	**1.008**	-.011	-.177	.054	.033	-.084	-.002
35	Cultural/religious events, celebrations are important for my life	**.720**	-.006	.040	-.097	-.026	.027	-.024
7	My health condition complicates the care of myself and my home	-.048	**.873**	-.098	.111	.084	.025	.003
5	I still have enough 5 strength and physical energy	-.067	**.795**	.053	.006	.070	-.247	.084
14	I am healthy enough 14 to have my independence	-.083	**.793**	.050	-.023	-.070	.026	-.111
8	I am healthy enough to get out and about	.042	**.668**	.087	-.074	-.058	.077	-.070
1	I enjoy my life overall	.007	**.624**	-.050	-.041	-.024	**-.619**	.031
32	I do paid (or unpaid) work or activities that I enjoy, it makes my life meaningful	-.014	**.615**	.123	-.013	-.088	-.013	-.061
6	Pain affects my wellbeing	-.039	**.611**	.010	.096	.135	-.086	-.033
30	I have hobbies and social activities that I enjoy doing	.047	**.519**	.015	-.069	.033	-.195	-.205
31	I try to stay involved in things and activities	.118	**.484**	-.033	-.160	-.009	-.071	-.252
4	Life gets me down	-.031	**.472**	-.120	-.220	.164	-.168	-.089
15	I enjoy whatever I do	-.041	**.467**	-.013	-.182	.021	-.055	-.309
33	I have to look after others which restricts my social or leisure activities	.043	**.418**	.003	.255	-.245	.100	.036
27	I have enough money to pay for household repairs or help in the house	.059	.013	**.877**	-.037	.009	.028	-.037
28	I can afford to buy what I want to	-.030	-.019	**.733**	-.092	.011	-.196	.079
26	I have enough money to pay household bills	.080	.075	**.684**	-.202	.032	.070	-.126
16	I cannot afford much from my pension, costs of living restrict my life	-.004	.065	**.495**	.097	.119	-.006	.004
29	I can’t afford to buy things I would enjoy	-.048	-.073	**.469**	.009	-.015	-.045	-.087
13	I have my children around which is important for me	-.028	-.047	-.035	**-.847**	-.009	.105	.051
9	My family, friends or neighbours would help me if need be	.080	.101	.031	**-.787**	.057	.062	.015
18	I feel safe where I live	.017	-.063	.026	**-.675**	.017	-.032	-.070
20	I feel fine in my home, neighbourhood and environment	.011	.019	.076	**-.558**	-.037	-.272	-.073
11	I have someone who gives me love and affection	.015	.087	.008	**-.549**	-.087	-.042	-.011
19	Local shops, services and other facilities are good overall	.014	.048	.115	**-.549**	-.035	-.033	.082
17	I can make decisions about my life	-.013	.230	-.021	**-.504**	.111	.054	-.182
21	I have friends around	.108	.009	.058	**-.434**	-.023	-.021	-.352
12	I would like to have more people around with whom I could enjoy my life	.050	.045	.018	-.169	**.888**	.040	.127
10	It would be better if I had more contact with people for a better social life	.070	-.011	.116	.036	**.685**	.034	.051
2	I feel happy most of the time	.021	**.480**	.081	-.020	.019	**-.560**	-.035
3	I enjoy my life	.039	**.504**	.022	-.011	-.077	**-.532**	-.082
22	I take my life as it comes and try to make the best of it	.005	.096	-.013	.023	.032	-.026	**-.896**
24	I tend to look on the bright side of the life	.074	-.022	.031	-.020	.049	-.135	**-.794**
25	If my health condition limits my social or leisure activities I try to compensate by finding something else I can do	-.049	.203	.008	-.031	-.181	.189	**-.602**
23	I feel happy compared to most people	.082	.191	.102	-.018	-.031	-.217	**-.559**

Note: Factor loadings higher than .4 are in bold. Matrix of polychoric correlation (two-step estimation) extracted by maximum-likelihood with Kaiser normalization and oblique Geomin rotation

**Table 4:** Correlation Matrix of Rotated Factors

	F1	F2	F3	F4	F5	F6	F7
F1	1.000	.063	.154	-.145	-.057	-.086	-.140
F2	.063	1.000	.375	-.488	.117	-.176	-.580
F3	.154	.375	1.000	-.369	.063	-.239	-.352
F4	-.145	-.488	-.369	1.000	.045	.386	.687
F5	-.057	.117	.063	.045	1.000	-.061	.044
F6	-.086	-.176	-.239	.386	-.061	1.000	.496
F7	-.140	-.580	-.352	.687	.044	.496	1.000

Note: Matrix of polychoric correlation (two-steps estimate) extracted by maximum-likelihood with Kaiser normalization and oblique Geomin rotation

We also point out also the item 34 with factor loading higher than one (1.008). As the pattern matrix contains standardized regression coefficients (not correlations) and factors were allowed to correlate, in the presence of high loadings in the structure matrix (in this case .978) coefficients higher than one are accepted without risk of negative residual variance [24].

There is also a problem with item 33 (“I have to look after others which restricts my social and leisure activities”). It seems to have only one and high loading on the second factor but in the structure matrix the highest loading (absolute value) did not exceed .231 and the item correlates just slightly with individual factors and also with the total scale (*r* = .068). For the reason of content validity and according to origin scale, we decided to leave the item in the questionnaire.

Items with the highest factor loading in the pattern matrix were used to construct domains of quality of life using item mean. Descriptives before standardization are listed in Table 5, all the scales had approximately normal distribution (analysed using P-P plots and histograms).

**Table 5:** Distribution of the Total Score and Individual Factors

		M	SD	Skewness	Kurtosis
1.	Role of belief, religion and culture	3.00	1.06	-.06	-.54
2.	Health, independence, active life	3.42	.69	-.61	.15
3.	Financial situation	3.03	.76	.11	.25
4.	Family and safe environment	2.91	.61	-.63	.90
5.	Loneliness	2.87	.81	.25	-.37
6.	Satisfaction with life	2.60	.80	-.71	.56
7.	Positive approach to life	2.74	.71	-.50	.14
Total score		121.33	18.00	-.43	.07

As these scores, based on the sum not on regression weights, have correlations slightly different from correlations of the original factors, we present them in Table 6.

**Table 6:** Correlation and reliabiliton of scale scores

	Number of factors	R	Role of belief, religion and culture	Health, independency, active life	Financial situation	Family and safe environment	Loneliness	Satisfaction with life	Positive approach to life	Total score
Role of belief, religion and culture	2	.78	1	.059	.097*	.152**	.010	.125**	.150**	.243**
Health, independency, active life	12	.91	.059	1	.419**	.578**	.085	.725**	.695**	.892**
Financial situation	5	.80	.097*	.419**	1	.422**	.139**	.427**	.419**	.645**
Family and safe environment	8	.85	.152**	.578**	.422**	1	-.007	.545**	.654**	.794**
Loneliness	2	.73	.010	.085	.139**	-.007	1	.043	-.055	.153**
Satisfaction with life	2	.83	.125**	.725**	.427**	.545**	.043	1	.680**	.784**
Positive approach to life	4	.85	.150**	.695**	.419**	.654**	-.055	.680**	1	.815**
Total score	35	.95	.243**	.892**	.645**	.794**	.153**	.784**	.815**	1

Note: r- internal consistency (Cronbach’s alpha, for the total score the composite reliability) * *p* < .05; ** *p* < .01

#### Reliability

We used the standardized Cronbach’s alpha for reliability estimation. Internal consistencies of scales are in a footer of Table 7 – it also contains corrected item-scales and item-total correlations. Internal consistencies are adequate, median of reliabilities of the dimensions is *Md*(*r*) = .830 (range .726–.905), reliability of the whole questionnaire .928 (Cronbach’s alpha).

**Table 7:** Corrected correlation of items with factors and the total score

		F1	F2	F3	F4	F5	F6	F7	Total score
1	I enjoy my life overall	.096	**.745**	.403	.561	.063	.884	.677	**.721**
2	I am happy most of the time	.133	.696	.458	.530	.104	**.831**	.668	**.704**
3	I enjoy life	.145	.697	.419	.556	.001	**.836**	.685	**.699**
4	Life gets me down	.053	**.629**	.296	.509	.171	.605	.539	**.600**
5	I still have enough strength and physical energy	-.034	**.786**	.382	.459	.196	.690	.541	**.669**
6	Pain does not allow me to feel well and comfortable	-.029	**.605**	.299	.281	.160	.420	.357	**.474**
7	My health condition complicates the care of myself and my home	-.045	**.720**	.211	.305	.165	.452	.386	**.512**
8	I am healthy enough to get out and about	.078	**.696**	.368	.465	.029	.554	.549	**.621**
9	My family, friends and neighbours help me if need be	.157	.493	.361	**.749**	.069	.467	.551	**.605**
10	It would be better if I had more contact with people for a more social life	.023	.026	.132	-.074	**.682**	.001	-.077	**.038**
11	I have someone who gives me love and affection	.142	.345	.275	**.560**	-.071	.409	.429	**.441**
12	I would like to have more people around with whom I could enjoy my life	-.003	.130	.141	.059	**.708**	.084	-.029	**.131**
13	I have my children around which is important for me	.051	.322	.260	**.634**	-.048	.308	.384	**.418**
14	I am healthy enough to have my independence	-.036	**.804**	.391	.511	.049	.643	.616	**.686**
15	I enjoy whatever I do	.074	**.741**	.390	.642	.071	.685	.744	**.733**
16	I can afford only little from my pension. costs of living restrict my life	.040	.229	**.500**	.129	.187	.174	.170	**.272**
17	I can make decisions about my life	.086	.550	.338	**.669**	.095	.452	.565	**.601**
18	I feel safe where I live	.108	.336	.290	**.655**	-.011	.349	.457	**.463**
19	Local shops. services and other facilities are good overall	.117	.343	.317	**.527**	.010	.305	.351	**.425**
20	I feel fine in my home. neighbourhood and environment	.165	.494	.420	**.720**	-.040	.601	.608	**.622**
21	I have friends in my neighbourhood	.218	.509	.377	**.674**	-.042	.527	.634	**.613**
22	I take my life as it comes and try to make the best of it-	.143	.652	.404	.645	-.029	.669	**.857**	**.709**
23	In comparison with other people I feel happy	.200	.659	.492	.642	-.022	.754	**.823**	**.738**
24	I tend to look on the bright side of life	.194	.580	.403	.621	-.004	.680	**.812**	**.671**
25	If my health condition limits my social or leisure activities I try to compensate by finding something else I am able to do	.067	.524	.258	.455	-.170	.427	.**622**	**.504**
26	I have enough money to pay household bills	.189	.505	**.774**	.560	.153	.528	.539	**.649**
27	I have enough money to pay for household repairs or help in the house	.151	.388	**.820**	.405	.156	.404	.411	**.526**
28	I can afford to buy what I want	.052	.331	**.782**	.394	.101	.407	.335	**.468**
29	I cannot afford to buy things I would like to have	-.001	.166	**.468**	.203	.060	.246	.219	**.260**
30	I have hobbies and social activities that I enjoy	.172	**.757**	.419	.560	.092	.661	.658	**.720**
31	I try to stay involved in various activities and to keep busy	.227	**.729**	.372	.592	.045	.627	.683	**.710**
32	I do paid (or unpaid) work or activities that I enjoy, it makes my life meaningful	.066	**.672**	.389	.421	.032	.521	.498	**.592**
33	I have to look after others which restricts my social or leisure activities	.007	.181	-.020	-.023	-.180	.073	.087	**.068**
34	Religion, belief, philosophy are important for my life	**.749**	-.014	.000	.080	.035	.074	.089	**.076**
35	Cultural/religious events and celebrations are important for my life	**.759**	.131	.203	.222	-.015	.182	.207	**.244**
	Number of items	2	12	5	8	2	2	4	**35**
	Standardized Cronbach’s alpha	.780	.905	.797	.850	.726	.830	.856	**.928**
	Reliability of linear combinations (composite scores)	-	-	-	-	-	-	-	**.950**

Note: Items in bold are in the same domain

This method of estimating the whole questionnaire reliability biased on multidimensional data usually leads to underestimation of reliability. Therefore we used composite score reliability of the sum of all dimensions (before standardization) using their reliabilities and covariances [31]. This estimation was higher, *r* = .950, and we consider it more accurate for the total score.

#### Validity

Table 8 includes parametric and nonparametric correlations of assessment of the overall quality of life with the individual domains and total score. Except for the first and fifth factors (Role of belief and religion, Loneliness) the correlations are moderate to strong, most correlations are statistically significant.

**Table 8:** Correlations of the overall quality of life with the individual domains

		Total score	F1	F2	F3	F4	F5	F6	F7
Overall quality of life	Pearson *r*	.705***	.074	.693***	.426***	.501***	.120***	.649***	.537***
	Kendall tau-b	.556	.076	.547	.341	.410	.090	.535	.421

Note: * *p* < .05; ** *p* < .01; *** *p* < .001

Furthermore, we verified the relationship of individual factors and overall score related to categorical variables: sex, age, marital status, current health state and type of housing. We used a factorial analysis of variance without interactions (they were not significant in any of the domains); the variables were included as “fixed-factors”. In all cases, the overall effect was significant, and for the significant predictors we then used the Bonferonni post-hoc test.

For domains of financial situation, *F*(13,454) = 5.21, *p < .*001 (η^2^ = .133), family and safe environment, *F*(13,454) = 7.05, *p* < .001 (η^2^ = .172), life satisfaction, *F*(13,454) = 7.55, *p* < .001 (η^2^ = .182), and positive approach, *F*(13,454) = 7.15, *p* < .001 (η^2^ = .174), the same predictors as in the case of total scores, *F*(13,454) = 16.14, *p* < .001 (η^2^ = .323) were significant; in addition, the said domains were related very closely to the total score (minimal correlation was *r*_*min*_ = .65). For this reason, we present the results of the ANOVA test only for the total score (Table 9). Marital status, housing and current health state had a significant effect on overall life satisfaction; relationship of the various categories, verified by post-hoc test, was similar and therefore we do not mention it.

**Table 9:** Factorial ANOVA for the total score of OPQOL-35 as the dependent variable

Source	Type III Sum of Squares	Df	Mean Square	F	Sig.	Partial Eta Squared
Model	146.73	13	11.259	16.136	.000	.323
Intercept	4.698	1	4.698	6.733	.010	.015
Sex	.069	1	.069	.099	.753	.000
Age	1.904	2	.952	1.365	.257	.006
Marital status	11.446	4	2.862	4.101	.003	.036
Housing	30.604	4	7.651	10.965	.000	.091
Current health state	31.968	2	15.984	22.907	.000	.094
Error	307.019	440	.698			
Total	453.396	454				

In all the domains, the healthy respondents were more satisfied with their quality of life than those with age-appropriate illnesses, and they in turn were more satisfied than the “fairly ill” respondents (all *p* < .001).

Married respondents are more satisfied than divorced or widowed ones (*p* < .001).

The exception is the domain “Belief, religion and culture”, in which on the contrary the less healthy respondents are happier.

Respondents who live alone at home, are self-sufficient and do not need the help of others, are more satisfied with their quality of life than all other respondents (all *p* < .001, apart from respondents living with their family, *p* < .05). Respondents who live alone with the help of their relatives or live with their family are more satisfied than respondents living in sheltered accommodation or nursing homes (all *p* < .001).

Monitored variables were also significantly related to the domain “Belief, religion and culture”, F(13, 454) = 2.24, *p* < .01, though the explained variance was relatively weak, η^2^ = .062 (see Table 10). Compared to the questionnaire as a whole, there was a slight significant difference between the sexes, men showing lower satisfaction (*p* < .05) in the questionnaire as a whole.

**Table 10:** Factorial ANOVA domains “Belief, religion and culture” as dependent variables

Source	Type III Sum of Squares	Df	Mean Square	F	Sig.	Partial Eta Squared
Corrected model	27.981	13	2.152	2.236	.008	.062
Intercept	1.887	1	1.887	1.960	.162	.004
Sex	4.747	1	4.747	4.932	.027	.011
Age	.796	2	.398	.413	.662	.002
Marital status	5.650	4	1.412	1.467	.211	.013
Housing	2.246	4	.562	.583	.675	.005
Current health state	14.535	2	7.267	7.551	.001	.033
Error	423.498	440	.962			
Total	451.479	454				

The impact of people’s health condition is opposite to that in the case of the questionnaire as a whole: healthy respondents achieve lower scores than those who do not describe their condition as healthy but as “age-appropriate” (*p* < .001), and also than the “fairly ill” seniors (*p* < .05). Interpretation of the findings is presented in the Discussion section.

The domain of “Loneliness”, F(13, 454) = 1.956, *p* < .05 has similar results although the effect here is even weaker, η^2^ = .055 (see Table 11). The only significant predictor for the degree of loneliness is the health condition, the effect of which is similar to the questionnaire as a whole – healthy respondents are less lonely than patients who are age-appropriately ill (*p* < .05) or the very sick ones (*p* < .01).

**Table 11:** Factorial ANOVA somains of loneliness as dependent variable

Source	Type III Sum of Squares	Df	Mean Square	F	Sig.	Partial Eta Squared
Corrected model	24.895	13	1.915	1.956	.023	.055
Intercept	.505	1	.505	.516	.473	.001
Sex	.489	1	.489	.500	.480	.001
Age	5.548	2	2.774	2.833	.060	.013
Marital status	3.949	4	.987	1.008	.403	.009
Housing	5.129	4	1.282	1.310	.266	.012
Current health state	9.466	2	4.733	4.834	.008	.021
Error	430.831	440	.979			
Total	455.734	454				

Related variables have the largest and a statistically significant effect F(13, 454) =27.253, *p* < .001 (see Table 12), on only one domain “Health, independence and active life” η^2^ = .446. It was indeed the dominant domain throughout the questionnaire, on which virtually all items had considerable factor loadings – this score may have thus the highest predictive validity. Apart from “Sex” all the predictors were significant.

**Table 12:** Factorial ANOVA domains “Health, independence, active life” as dependent variables

Source	Type III Sum of Squares	Df	Mean Square	F	Sig.	Partial Eta Squared
Corrected model	202.139	13	15.549	27.253	.000	.446
Intercept	1.519	1	1.519	2.662	.103	.006
Sex	.064	1	.064	.112	.738	.000
Age	6.371	2	3.185	5.583	.004	.025
Family status	9.091	4	2.273	3.983	.003	.035
Housing	31.370	4	7.843	13.746	.000	.111
Current health state	50.163	2	25.081	43.960	.000	.167
Error	251.040	440	.571			
Total	453.183	454				

Satisfaction with their “Health, independence and active life” significantly decreases with age, older age groups are always less satisfied (each *p* < .001).

Single respondents (*p* < .01), married ones (*p* < .001) and respondents with a partner (*p* < .05) were significantly more satisfied than widowed respondents. Married respondents are also more satisfied than the divorced ones (*p* < .01).

Furthermore, respondents living independently at home are happier than all other respondents (*p* < .001). Conversely, respondents living in retirement homes are less happy than all the others (*p* < .001, in the case of nursing homes *p* < .05). Respondents living at home, with their family or in nursing homes do not differ (*p* > .05).

Then in conclusion, the feeling of being content in this domain is declining according to the state of health – the healthy respondents are more satisfied than patients with age-appropriate illnesses, and those again are more satisfied than the very ill ones (all *p* < .001).

## Discussion

The Czech version of the OPQOL-35 questionnaire – just like the original version – is designed to assess the quality of life of seniors. It understands the quality of life as a multidimensional entity, so it can determine, on the basis of the results obtained, in which domains seniors do not differ from their peers, in which they get on better, and in which domains, on the contrary, they experience difficulties that must be addressed.

When translating the English text we took into account some socio-cultural specificities and the conditions under which Czech seniors live. Based on the pilot testing of the questionnaire, we changed the assessment scale for the answers. Instead of assigning points we offered them a five-point grading which is common in Czech schools, i.e. 1 = strongly agree, 5 = strongly disagree.

Czech factor analyses of Czech data showed that the questionnaire items grouped somewhat differently from the original version, so that instead of 8 domains we had 7 domains that are in this respect well interpretable. Seven extracted factors explained 62.3 % of the variance. This result is better than that of Bowling’s study [16, 21] which, using the method of principal components, extracted nine factors of 60.6 % of explained variance. In our data, the initial variance (which would have corresponded to principal component analysis) was explained by the first nine factors of 75.0 %.

We discovered a discrepancy in the item no. 33 (“I have to take care of others, which limits my social or leisure activities”), between the factual importance of the item and the results of item analysis. Seniors, spouses or elderly family members, are often caring for a family member who is not fully self-reliant (usually also a senior), which means there is little time left to themselves. We have therefore decided to leave that item in the questionnaire as it is important to determine the specific type of load on the senior, and it can signal the possibility of targeted interventions.

Anyway, the original eight-factor structure was based on open-ended survey [21], but its principal component analyses revealed two [22], four [22] or nine-factors [18] solutions; PCA of Chinese version extracted eight factors similar to the original version [23]. We can conclude that the factor structure of OPQOL is probably unclear also in the original version, and we suggest its thorough confirmation.

Moreover, it seems that original eight factors represent more domains of life than latent factors underlying the quality of life, and thus they are not appropriate for clinical interpretation.

They don’t fit data in our confirmatory factor analysis either, nor in the original principal component analyses [18, 22]. Our exploratory factor solution can represent these underlying factors.

Unfortunately, three of our factors contain only two items with the highest loadings, and so the factor structure is not probably stable enough – factors “Loneliness” and “Satisfaction with Life” could be probably incorporated into some other factors. In our version of OPQOL questionnaire we suggest to drop out one of two items in those factors, which could lead to more stable structure with smaller number of factors, or, alternatively, add some more items into those factors. In any case, a confirmatory factor analysis of the Czech version (and original English version, too) in a different sample is needed.

The internal consistencies of all scales were satisfactory, ranging from .726 to .905, which was similar to the original version. The reliability of the whole questionnaire was .950. We conclude that OPQOL is a reliable criterion of perceived quality of life.

We confirmed the findings of other authors that sex, age, marital status, current state of health and type of housing affect the perceived quality of life of the elderly.

We noted that in the domain of “Belief, religion and culture” more ill seniors reported they were happier than healthy people. This paradoxical finding can be interpreted that if seniors are religious people, they take their illness as a test of their “strength in faith” (as opposed to seniors who are non-believers) and they also trust in their God, whose social support surpasses human capabilities. Their hope is not small, prosaic, horizontal, but a hope from which they draw the deepest securities in life, the supreme values of life – vertical hopes.

Our data also showed that respondents, who live alone at home and do not need the help of others, are more satisfied with their quality of life than all other respondents. Research of [32] into 10 Italian senior citizens living alone and seniors living with others, brought somewhat different results: independent of demographic, socioeconomic, functional and clinical characteristics, quality of life of older people living alone at home is similar to quality of life of older adults living with others. Two specific dimensions of quality of life were worse in older people living alone: “social relationships” and “home and neighbourhood”. The author explains the worse quality of life of the elderly in these two dimensions as follows: depression, having no caregiver and never having been married were the characteristics independently associated with a poor quality of life amongst older outpatients living alone.

Said contradiction of the positive effects of the disease can be explained according to Albrecht and Devlieger by the theory of equilibrium: many people with serious illness can create a balance between the physical, mental and spiritual components of their lives [33].

## Conclusions

Assessment of the quality of life of seniors is important for adequate provision of both health and social care. There are a number of diagnostic methods for determining the quality of life of seniors, but for routine use the generic questionnaires are probably the most appropriate ones. The result of our work was the creation of the Czech version of the British questionnaire *Older People’s Quality of Life Questionnaire* (OPQOL-35). Unlike the British version, which has eight factors, we had a seven factor solution. Individual factors are named as follows: 1 Role of belief, religion and culture; 2 Health; independence, active life; 3 Financial situation; 4 Family and safe environment; 5 Loneliness; 6 Satisfaction with life; 7 Positive approach to life. The OPQOL-CZ-35 questionnaire has a very good reliability (Cronbach’s alpha ranging from .726 to .905); the reliability of the questionnaire as a whole is .950.

The Czech version is also satisfactorily valid. Our results show that with increasing age and number of health problems the satisfaction of respondents is generally declining in all seven domains. Conversely, the degree of independence concerning housing is positively associated with satisfaction. Single persons (although due to small numbers of single respondents the differences were not significant) and married respondents are generally happier in some domains than the divorced and widowed ones.

It seems that for differential diagnosis the overall score (and optionally the domain “Health, independence and active life”) is more useful. Other domains functioned very similarly, achieving high mutual correlations. The exception is the domain of “Belief, religion and culture”, in which, on the contrary, more ill respondents are happier, and which has relatively low correlations with other domains. Low correlation with other factors has also the domain “Loneliness” although it may be of clinical significance. However, it contains only two items (in the same way, however, as “Belief, religion and culture”) and is little related to other controlled variables (significant relationship was found only in the cases of health/illness state).

Experience with the use of the OPQOL-35-E questionnaire shows that for most seniors 35 items are relatively too many. To use the questionnaire in routine clinical practice it is necessary to create a shorter version, as done by the team led by A. Bowling [34]. We are working on a shortened Czech version as well.

## Abbreviations

ASCOT, adult social care outcomes toolkit; CASP-19, control, autonomy, self-regulation, Pleasure; OC, optimal coordinates; OPQOL - 35, older people’s quality of life - 35; PA, parallel-analysis; QUAL-E, quality of life at the end of Life; SEIQOL, Schedule for the Evaluation of Individual Quality of Life; WHOQOL-OLD, World Health Organization Quality of Life-Old
